# Influence of Vitamin E Supplementation on Glycaemic Control: A Meta-Analysis of Randomised Controlled Trials

**DOI:** 10.1371/journal.pone.0095008

**Published:** 2014-04-16

**Authors:** Renfan Xu, Shasha Zhang, Anyu Tao, Guangzhi Chen, Muxun Zhang

**Affiliations:** 1 Department of Endocrinology, Tongji Hospital, Tongji Medical College, Huazhong University of Science and Technology, Wuhan, People's Republic of China; 2 Department of Internal Medicine and Gene Therapy Center, Tongji Hospital, Tongji Medical College, Huazhong University of Science and Technology, Wuhan, People's Republic of China; 3 Department of Medical Ultrasound, Tongji Hospital, Tongji Medical College, Huazhong University of Science and Technology, Wuhan, People's Republic of China; University College Dublin, Ireland

## Abstract

Observational studies have revealed that higher serum vitamin E concentrations and increased vitamin E intake and vitamin E supplementation are associated with beneficial effects on glycaemic control in type 2 diabetes mellitus (T2DM). However, whether vitamin E supplementation exerts a definitive effect on glycaemic control remains unclear. This article involves a meta-analysis of randomised controlled trials of vitamin E to better characterise its impact on HbA1c, fasting glucose and fasting insulin. PubMed, EMBASE and the Cochrane Library were electronically searched from the earliest possible date through April 2013 for all relevant studies. Weighted mean difference (WMD) was calculated for net changes using fixed-effects or random-effects models. Standard methods for assessing statistical heterogeneity and publication bias were used. Fourteen randomised controlled trials involving individual data on 714 subjects were collected in this meta-analysis. Increased vitamin E supplementation did not result in significant benefits in glycaemic control as measured by reductions in HbA1c, fasting glucose and fasting insulin. Subgroup analyses revealed a significant reduction in HbA1c (−0.58%, 95% CI −0.83 to −0.34) and fasting insulin (−9.0 pmol/l, 95% CI −15.90 to −2.10) compared with controls in patients with low baseline vitamin E status. Subgroup analyses also demonstrated that the outcomes may have been influenced by the vitamin E dosage, study duration, ethnic group, serum HbA1c concentration, and fasting glucose control status. In conclusion, there is currently insufficient evidence to support a potential beneficial effect of vitamin E supplementation on improvements of HbA1c and fasting glucose and insulin concentrations in subjects with T2DM.

## Introduction

Type 2 diabetes mellitus (T2DM) is a global health problem affecting almost 336 million people worldwide or approximately 8.3% of the world population, and the number of affected individuals will dramatically increase in the next 20 years [1]. Poor glycaemic control is responsible for the long-term negative outcomes in T2DM subjects, including microvascular and macrovascular complications, such as cardiovascular events, renal failure, blindness and peripheral neuropathy [2]. Appropriate management of hyperglycaemia is needed to reduce morbidity and the number of complications associated with T2DM.

The association between oxidative stress and T2DM has long been recognised and is based on the observation that hyperglycaemia, hyperinsulinaemia, and insulin resistance can enhance free radical generation and thus contribute to oxidative stress [3]. Oxidative stress can in turn promote haemoglobin glycation [4], [5] and impair insulin signalling and β-cell insulin secretion in T2DM [6], [7]. Thus, it is reasonable to postulate that antioxidants, such as vitamin E, may have benefits effects on glycaemic control in T2DM.

Vitamin E is a powerful lipid-soluble antioxidant that reduces oxidative stress and decreases the oxidative stress-associated damage in T2DM [8]. Anti-hyperglycaemic effects of vitamin E have been hypothesised, tested in laboratory and human studies, and have gained biological plausibility. Observational studies have indicated that vitamin E intake was inversely related to a risk of T2DM [9]. Furthermore, in patients with established T2DM, regular vitamin E supplementation is associated with a significant improvement in glycaemic control [10], which provide justification for trials evaluating vitamin E supplementation and glycaemic control in T2DM subjects.

There are several possible mechanisms underlying the association between vitamin E and glucose metabolism. Vitamin E may prevent the glycosylation of haemoglobin by inhibiting the formation of advanced glycosylation endproducts (AGEs) [11]. Vitamin E can also mitigate the long-term pancreatic β-cell dysfunction caused by oxidative stress in T2DM [12]. Vitamin E supplementation was also found to be associated with other disorders, including Nonalcoholic Steatohepatitis [13] and age-related cataract [14], while there is no evidence to support the use of vitamin E supplements for prevention of cardiovascular diseases [15] and stroke [16].

Vitamin E (tocopherols and tocotrienols) is an essential micronutrient that is acquired primarily through the consumption of fruit, vegetable oils, nuts, and green leafy vegetables [17]. In addition, vitamin E capsules are readily available and often supplied for healthy people, and approximately 12.7% of the adult population in the United States takes vitamin E as dietary supplements [18]. Given that vitamin E intake is simple, safe, and inexpensive, the vitamin E supplementation may represent a common and easily obtainable treatment for glycaemic control.

The objective of this meta-analysis was to combine evidence from randomised controlled trials (RCTs) to assess the effect of vitamin E supplementation on change in HbA1c, a well-established clinical marker of long-term glycaemic control, and on fasting glucose and fasting insulin levels in subjects with T2DM.

## Experimental Methods

### Search selection

We searched the PubMed(http://www.ncbi.nlm.nih.gov/pubmed/), EMBASE(http://www.embase.com/) and Cochrane library databases (http://www.cochrane.org/) from their inception until April 2013 for trials that examined the effects of vitamin E on HbA1c, glucose control and insulin sensitivity in humans. The following search terms were used: (vitamin E OR alpha-tocopherol OR tocopherols OR tocotrienols) AND (glucose OR hyperglycaemia OR glyc(a)emia OR insulin OR insulin sensitivity OR insulin resistance OR HbA1c OR glycated protein or glycated albumin OR fructosamine OR diabetes). The search was confined to human studies. Manual recursive searches were also conducted on references from review articles and published randomised controlled trials that met the inclusion criteria. There was no restriction by language or publication status.

### Study selection

Potentially relevant studies were selected based on the following inclusion criteria: (1) randomised controlled trials with either a parallel or a crossover design; (2) adults with T2DM supplied with a vitamin E supplementation that is currently FDA-approved for at least 6 weeks treatment; (3) fasting glucose, fasting insulin, HbA1c or glycated haemoglobin levels were describes in the clinical trials; And (4) studies used a concurrent control group, and the difference between the treatment and the control groups was limited to vitamin E. Exclusion criteria were as follows: (1) trials that enrolled children, pregnant women, or patients with type 1 diabetes; (2) patients were supplied with both vitamin E and other antioxidants; and (3) trials with abstracts only.

### Data extraction

Two investigators independently selected the trials and extracted the data, and disagreements or uncertainties were resolved by consensus. Extracted data included study characteristics (author, publication status, year, design), treatment characteristics (type of intervention, dosage, frequency, duration), and participant characteristics (sample, mean age, gender, race/ethnicity, serum vitamin E level, mean HbA1c and fasting glucose levels). Primary outcome measures included the net changes in plasma concentrations of glycated haemoglobin (HbA1c), fasting glucose levels, and fasting insulin concentrations after vitamin E supplementation. The secondary outcome included changes in the homeostatic model assessment of insulin resistance (HOMA-IR), postprandial glucose and insulin concentrations, total cholesterol (TC), triglycerides (TG), high density lipoprotein cholesterol (HDL-c), low density lipoprotein cholesterol (LDL-c), systolic blood pressure (SBP) and diastolic blood pressure (DBP).

### Quality assessment

The quality of the included studies was assessed using the Jadad scoring system [19]. Trials scored one point for each area addressed in the study (randomisation, double-blinding, description of withdrawals and dropouts, generation of random numbers and allocation concealment), with a possible score between 0 and 5. Trials were subclassified as high-quality (Jadad score ≥4) or low-quality (Jadad score of 2 or 3).

### Statistical analyses

The statistical analysis was performed with RevMan software version 5 (Cochrane Collaboration). The mean change in the outcome variables from baseline was treated as a continuous variable, and the weighted mean difference was calculated as the difference between the mean in the vitamin E group and control groups. The significance of any mean changes was evaluated using the WMD and 95% CIs with fixed-effect or random-effect models. If the variances for the mean changes were not reported directly, the variances were imputed from the exact P value, CIs, or individual variances from the vitamin E group and the control group [20]. Missing variances for paired differences were imputed by assuming a correlation coefficient of 0.5 between variances at baselines and ends of trials according to the method of Follmann et al [21]. We assumed equal variances during the trial and between intervention and control groups.

Heterogeneity was assessed using the Cochrane Q statistic and the inconsistency index (I^2^), where a P value<0.10 was considered significant, and measured in-consistency (I^2^) statistics, with a measurement >50% taken to indicate substantial heterogeneity [22]. A random-effect model was used to analyse the results with significant heterogeneity. Otherwise, a fixed-effect model was used for outcomes without obvious heterogeneity [23]. Publication bias was assessed using funnel plots and Egger's test [24]. To explore the possible influence of covariates on net changes, subgroup analyses were conducted to evaluate the effects of the following factors on primary outcomes (HbA1c, fasting glucose and insulin concentrations): vitamin E dosages, ethnic groups, vitamin E duration, baseline HbA1c concentrations, baseline fasting glucose concentrations and the baseline serum vitamin E concentrations of the participants. A P value <0.05 was considered to be statistically significant in this trial, unless otherwise specified.

## Results

### Literature search

**Figure 1:**
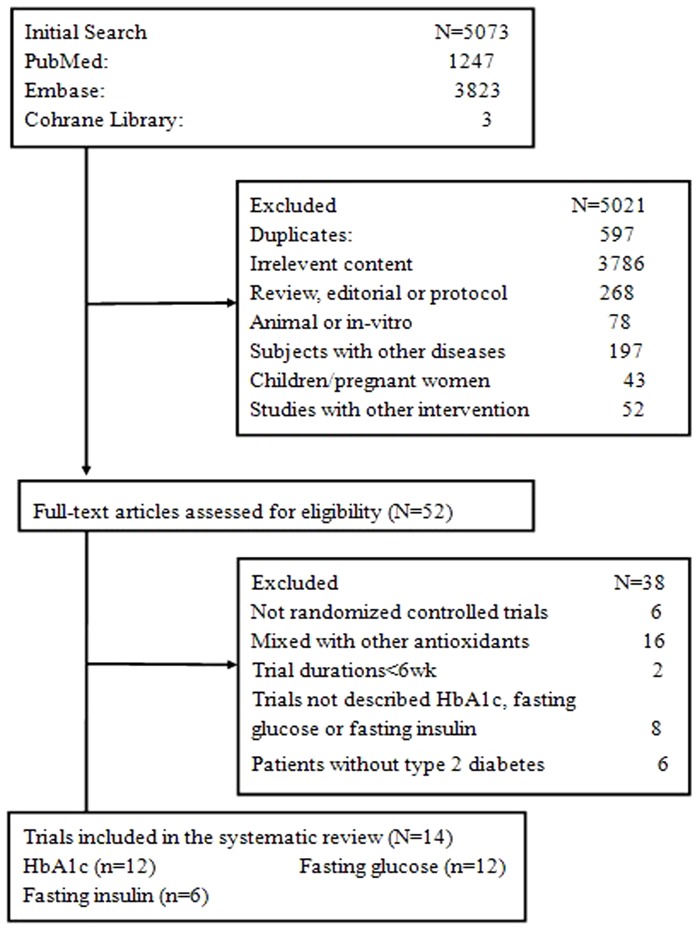
Flow diagram of the process of article selection for meta-analysis.

We identified 5073 potentially relevant articles from the PubMed, EMBASE and Cochrane library databases, 5021 of which were determined to be irrelevant after review of the titles and abstracts. 52 trials proceeded to a detailed evaluation of the complete report, and a further 38 were excluded. A total of 14 trials met the selection criteria for the current meta-analysis. A flow describing article selection for this meta-analysis is shown in Figure 1.

### Study description

Fourteen published studies with a total of 714 participants, including 363 subjects in the vitamin E group and 351 patients in the control group, were identified in this meta-analysis [25]–[38]. The characteristics of the studies and participants are outlined in Table 1. Three primary outcomes measures were examined: HbA1c (12 studies) [25]–[36], fasting glucose (12 studies) [25]–[31], [34], [36]–[38] and fasting insulin (6 studies) [29]–[31], [36]–[38]. Among them, one trial examined the effect of vitamin E in both type 1 and type 2 diabetes; only groups with type 2 diabetes were selected for each run of analysis [33]. Twelve of the fourteen included studies were exhibited a parallel design [26]–[34], [36]–[38], whereas the other two were crossover studies [25], [35]. The dosage of vitamin E supplements ranged from 200 to 1600 IU/d, with an intervention duration ranged from 6 to 27 weeks. Nine studies focused on subjects in western countries [25], [26], [28]–[30], [33], [34], [37], [38] and five studies focused on subjects in Asian countries [27], [31], [32], [35], [36]. Most studies enrolled patients with normal serum vitamin E levels [25]–[29], [31]–[35], [37], whereas three other studies enrolled subjects with low serum vitamin E levels [30], [36], [38]. The participants in eight studies exhibited poor fasting glucose control (>8.0 mmol/L) at baseline [25], [26], [28]–[31], [34], [36], and the other four trials exhibited good fasting glucose control at baseline [27], [35], [37], [38]. Four studies exhibited high serum HbA1c (>8.0) levels at baseline [30], [31], [34], [36], whereas the other eight studies exhibited well-controlled serum HbA1c (<8.0) levels at baseline [25]–[29], [32], [33], [35].

**Table 1:** Characteristic of experimental trials included in the meta-analysis.

Trial	Country	Vitamin E group intervention	Control group	Duration weeks(n)	Study Size(n)		Mean age (year)		Male(%)		Diabetes duration(years)		Trial design	Baseline HbA1c(%)	Baseline FPG(mmol/l)	Baseline Vit E (mmol/l)
					T	C	T	C	T	C	T	C				
Paolisso 1993	Italy	d-α-tocopherol 900 mg/d	Placebo	12	25	25	71.3	71.3	—	—	8.4	8.4	C1	7.0	8.2	16.3
Reaven 1995	USA	dl-α-tocopherol 1600 IU/d	Placebo	10	10	11	60.8	61.8	100	100	8.6	8.3	P	7.7	9.9	35
Tutuncu 1998	Turkey	dl-α-tocopherol acetate 900 mg/d	Placebo	24	11	10	51.2	59.3	18.2	10	8.8	8.9	P	7.5	7.2	—
Gazis 1999	UK	α-tocopherol 1600 IU/d	Placebo	8	23	25	56.4	57.9	65.2	84	4.3	4.8	P	6.9	9.6	27.8
Paolisso 2000	Italy	Vitamin E 600 mg	Placebo	8	20	20	58.3	56.7	45	60	7.8	7.8	P	7.7	8.3	16.9
Manzella 2001	Italy	All-rac-α-tocopherolAcetate 600 mg/d	Sodium citrate	16	25	25	64.3	65.1	—	—	—	—	P	8.2	9.1	7.1
Park 2002	Korea	α-tocopherol 200 mg/d	Digestive pill	8	48	50	49.4	49.5	56.3	62.8	7.9	8.3	P	10.9	10.4	25.5
Chen 2004	China	Vitamin E 200 mg	Placebo	52	41	41	52	52.4	51.2	48.8	<1	<1	P	7.6	—	—
Economides 2005	USA	All-rac-α-tocopherol Acetate 1800 IU/d	Soybeanoil	24	25	20	59	59	56.7	56.7	9	9	P	7.2	—	28.9
Ble-Castillo 2005	Mexico	α-tocopherol 800 IU/d	Corn strach	6	13	21	51.3	55.3	0	0	<14	<14	P	10.9	12.8	—
Baliarsingh 2005	India	Tocotrienols 6 mg/kg	Rice brain oil	8	10	9	48.5	52.6	50	55.6	5.3	4.3	C1	7.6	6.3	—
Boshtam 2005	Iran	Vitamin E 200 IU/d	Placebo	27	50	50	52.8	54.5	—	—	5.7	13.3	P	9.9	10.8	10.5
Ward 2007	Australia	RRR-α-tocopherol or mixd tocopherols 500 mg/d	Soy bean oil	6	37	18	61	62	67.5	88.9	—	—	P	—	7.3	—
de Oliveira 2011	Brazil	α-tocopherol 800 IU/d	Placebo	16	25	26	62	63	72	57.7	—	—	P	—	7.2	4.7

FPG: fasting plasma glucose; vitE: vitamin E; mixed tocopherols: 60%γ-,25% δ- and 15% α-tocopherol; HbA1c: glycated hemoglobin; USA: United States of America; UK: The United Kingdom; T:treatment group; C: control group; C1: crossover design; P: Parallel design.

### Primary outcomes

**Figure 2:**
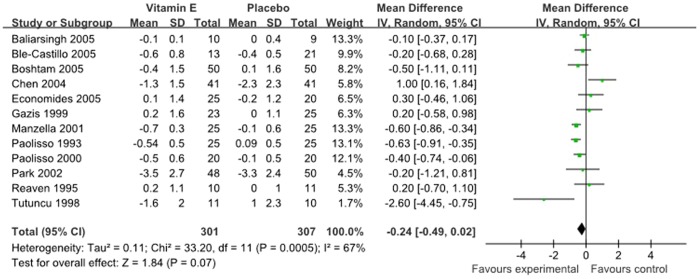
Forest plot of randomised controlled trials investigate the effect of vitamin E supplementation on HbA1c.

Primary outcome measures included changes in HbA1c, fasting glucose and fasting insulin. Twelve studies with 608 subjects, including 301 subjects in vitamin E group and 307 subjects in placebo group, reported HbA1c at baseline and follow-up. There was no significant reduction in the percentage of HbA1c (−0.24, 95% CI: −0.49 to 0.02]; P = 0.07, Figure 2), with significant evidence of interstudy heterogeneity (I^2^ = 67%; P = 0.0005) in subjects with vitamin E supplements, so we reported the results from random-effects models.

**Figure 3:**
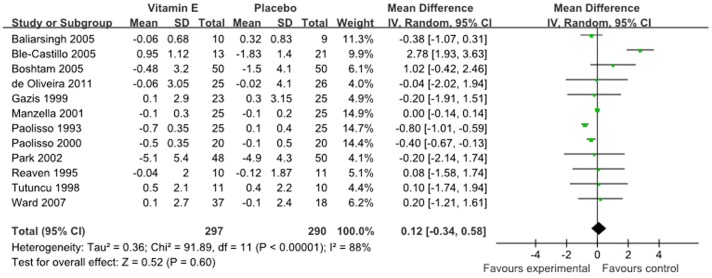
Forest plot of randomised controlled trials investigate the effect of vitamin E supplementation on fasting glucose.

The results for fasting glucose were reported in 12 studies representing 587 participants, including 290 controls. Meta-analyses suggested that vitamin E supplementation, compared with a placebo or control, resulted in no statistically significant improvement (0.12 mmol/l, 95% CI: −0.34 to 0.58 mmol/l, P = 0.60, Figure 3) in fasting glucose status. The degree of heterogeneity was significant (I^2^ = 88%; P<0.00001), and random-effects models were performed in this meta-analysis.

**Figure 4:**
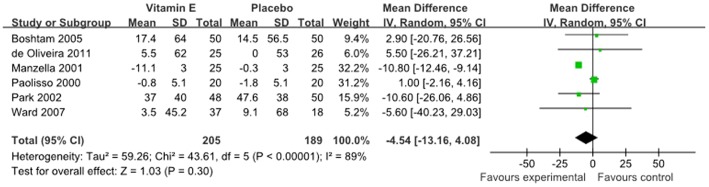
Forest plot of randomised controlled trials investigate the effect of vitamin E supplementation on fasting insulin.

The mean change in fasting insulin concentrations was reported in 6 studies, representing 394 participants, and was not significantly decreased in the intervention groups (−4.54 pmol/l, 95%CI: −13.16 to 4.08 pmol/l, P = 0.30, Figure 4) compared to control arms. Significant heterogeneity (I^2^ = 89%; P<0.00001) was observed, and the results are reported on the basis of random-effects models.

### Subgroup analysis

Subgroup analyses were conducted to explore the dose-effect relationship, study-duration effects, ethnic group variation, and differences in baseline HbA1c, fasting glucose and serum vitamin E concentrations. The results are summarised in Table 2.

**Table 2:** Subgroup analyses of HbA1c, fasting glucose and fasting insulin stratified by previously defined study characteristics.

Variables	HbA1c(%)			Fasting glucose(mmol/l)			Fasting insulin(pmol/l)		
	No.of tirals	Mean difference(95%CI)	P for heterogeneity	No.of tirals	Mean difference(95%CI)	P for heterogeneity	No.of tirals	Mean difference(95%CI)	P for heterogeneity
Subgroup analysis									
Vitamin E dose									
≤400 mg/d	4	−0.0(−0.52,0.51)	0.04	3	0.01(−0.85,0.87)	0.23	2	−6.56(−19.50,6.38)	0.35
>400 mg/d	8	−0.35(−0.63,−0.07)	0.01	9	0.15(−0.38,0.68)	<0.0001	4	−4.13(−14.48,6.21)	<0.0001
Duration									
Shorter term (<12 wk)	6	−0.17(−0.35,0.01)	0.62	7	0.31(−0.68,1.29)	<0.001	3	0.33(−5.49,4.82)	0.33
Longer term (≥12 wk)	6	−0.33(−0.79,0.13)	0.0004	5	−0.14(−0.78,0.50)	<0.001	3	−9.0(−15.90, −2.10)	0.32
BaselineHbA1c (%)									
<8	8	−0.12(−0.49,0.25)	0.0002	6	−0.55(−0.81, −0.29)	0.19	1	1.00(−2.16,4.16)	-
≥8	4	−0.49(−0.71, −0.28)	0.5	4	0.95(−0.70,2.59)	<0.001	5	−10.67(−12.31, −9.02)	0.64
Baseline fasting glucose (mmol/l)									
<8	2	−1.18(−3.60,1.25)	0.009	4	−0.22(−0.78,0.35)	0.87	2	0.44(−22.95,23.82)	0.64
≥8	8	−0.44(−0.62, −0.26)	0.27	8	0.21(−0.34,0.77)	<0.0001	4	−5.15(−14.48,4.18)	<0.0001
Ethnicity									
Asian	5	−0.21(−0.84,0.43)	0.004	4	−0.10(−0.67,0.46)	0.39	2	−6.56(−19.50,6.38)	0.35
Western	7	−0.34(−0.58, −0.09)	0.05	8	0.15(−0.4,0.7)	<0.0001	4	−4.13(−14.48,6.21)	<0.0001
Baseline serum Vitamin E(mmol/l)									
Normal	6	−0.23(−0.56,0.09)	0.08	5	−0.57(−0.87, −0.28)	0.16	2	−2.23(−12.42,7.96)	0.15
Low	2	−0.58(−0.83, −0.34)	0.77	3	0.01(−0.13,0.15)	0.39	3	−9.0(−15.90, −2.10)	0.32

In general, the reduction in HbA1c and fasting insulin were greater in studies with low serum vitamin E concentrations, with mean changes of −0.58 (95% CI: −0.83, −0.34) and −9.0 pmol/l (95% CI: −15.90, −2.10 pmol/l) respectively. In contrast, fasting glucose decreased significantly in studies with normal vitamin E concentrations (−0.57 mmol/l; 95% CI: −0.87, −0.28 mmol/l). Trials with low serum vitamin E concentrations were limited in this meta-analysis, so more trials are needed in the following research area.

Subgroup analysis also suggested that HbA1c and fasting insulin were reduced to a greater degree in studies with higher HbA1c concentrations (≥8%), with mean changes of −0.49 (95% CI: −0.71, −0.28) and −10.67 pmol/l (95% CI: −12.31, −9.02 pmol/l). In contrast, there was a negative dose-response relationship between HbA1c concentrations (<8%) and the reduction in fasting glucose. Significant reductions in HbA1c were also observed in studies with poor fasting glucose concentrations (≥8 mmol/l) (−0.44 mmol/l; 95% CI: −0.62, −0.26 mmol/l).

A significant reduction in HbA1c was observed in studies with higher vitamin E doses (>400 mg/d), with a net change of −0.35 (95% CI: −0.63, −0.07). Similarly, concentrations of fasting insulin declined significantly in studies with longer vitamin E treatment durations (>12 wk) (−9.0 pmol/l; 95% CI: −15.90, −2.10 pmol/l). Western population with vitamin supplementation also yielded significant effect on HbA1c (−0.34; 95% CI: −0.58, −0.09) compared with controls. In conclusion, although there are significant results in subgroup analysis, more trials with larger subjects are needed to make a definite conclusion.

A sensitivity analysis was performed by excluding three trials (Economides 2005, Baliarsingh 2005, Ward 2007) which used an oil for placebo. The exclusion of trials resulted in greater reductions in HbA1c (−0.30, 95% CI: −0.59, −0.02; P = 0.04), which significantly influenced the results. While, after exclusion these trials, the pooled reductions in fasting glucose and fasting insulin were not significantly influenced, with 0.19 mmol/l (95% CI: −0.33, 0.71 mmol/l; P = 0.47) and −4.33 pmol/l (95% CI: −13.06, 4.41 pmol/l; P = 0.33), respectively. In a sensitivity analysis, after exclusion the trial (Park 2002) with digestive pill, the pooled reductions in HbA1c, fasting glucose and fasting insulin were not significantly influenced, with −0.24 (95% CI: −0.50, 0.03; P = 0.08), 0.12 mmol/l (95% CI: −0.35, 0.59 mmol/l; P = 0.62) and −3.06 pmol/l (95% CI: −12.82, 6.70 pmol/l; P = 0.54), respectively.

### Secondary outcomes

Pooled analysis of the changes in secondary outcomes was performed by calculating or estimating the weighted averages. These changes are summarised in Tables 3. Vitamin E resulted in no significant decrease in HOMA-IR (−0.32, 95% CI:−0.65 to 0.01, P = 0.06) or postprandial glucose (−0.20, 95% CI: −1.07 to 0.67, P = 0.65) compared with controls. Vitamin E supplementation also appeared to have no statistically significant effect on lipid metabolism, including triglyceride (TG), total cholesterol (TC), low-density lipoprotein-cholesterol (LDL-C), and high-density lipoprotein-cholesterol (HDL-C), compared with placebo. Furthermore, vitamin E supplementation failed to significantly alter either systolic blood pressure (SBP) or diastolic blood pressure (DBP) compared with the control group.

**Table 3:** Summary of effect sizes (weighted mean difference) for secondary outcomes.

Variables	No. of comparison	Sample size		Net change(95%CI)	P	Test for heterogenenity	
		T	C			I^2^(%)	P
HOMA index	3	70	71	−0.32(−0.65,0.01)	0.06	89	0.0001
Postprandial glucose	3	69	69	−0.20(−1.07,0.67)	0.65	0	0.77
Triglyceride(mmol/l)	8	201	212	−0.02(−0.29,0.25)	0.90	96	<0.0001
Total-C(mmol/l)	9	254	252	−0.29(−0.85,0.27)	0.31	99	<0.0001
HDL-c(mmol/l)	9	217	223	−0.00(−0.03,0.02)	0.70	20	0.27
LDL-c(mmol/l)	8	192	203	−0.24(−0.78,0.29)	0.37	98	<0.0001

HOMA, homeostasis model assessment; Total-C, total cholesterol; HDL-C, high-density lipoprotein-cholesterol;LDL-C, low-density lipoprotein-cholesterol; CI, confidence interval. T: treatment group; C: control group.

### Study quality and publication bias

The quality of these fourteen randomised controlled trials was variable (Table 4). Only one study was classified as high quality (Jadad score of 4) [37], and thirteen studies were classified as low quality (Jadad score of 2 or 3) [25]–[36], [38]. Randomisation was a prerequisite for inclusion in this meta-analysis, so all studies were randomised, but only two trails mentioned the method for sequence generation [32], [37]. Thirteen studies were double-blind [25]–[31], [33]–[38], and one study did not describe blinding [32]. Ten trials presented a clear explanation for withdrawals and dropouts in each group [25], [26], [28], [29], [31]–[35], [37].

**Table 4:** Quality assessment of included studies.

Authors	Randomisation	Allocation concealment	Random sequence generation	Blinding	Reporing of withdrawals	Jaded score
Paolisso 1993	Y	U	U	Y	Y	3
Reaven 1995	Y	U	U	Y	Y	3
Tutuncu 1998	Y	U	U	Y	U	2
Gazis 1999	Y	U	U	Y	Y	3
Paolisso 2000	Y	U	U	Y	Y	3
Manzella 2001	Y	U	U	Y	U	2
Park 2002	Y	U	U	Y	Y	3
Chen 2004	Y	U	Y	U	Y	3
Economides 2005	Y	U	U	Y	Y	3
Ble-Castillo 2005	Y	U	U	Y	Y	3
Baliarsingh 2005	Y	U	U	Y	Y	3
Boshtam 2005	Y	U	U	Y	U	2
Ward 2007	Y	U	Y	Y	Y	4
Oliveira 2011	Y	U	U	Y	U	2

Y, yes; U, unclear; Randomisation:the study described as randomized; Random sequence generation: the correct method for generation of random numbers computer random numbers table, shuffled cards or tossed coins, and minimization; Allocation concealment: Adequate concealment was that up to the point of treatment (eg, central randomisation); Double-blinding: masking to both researchers and patients; Reporting of withdrawals: The numbers and reasons for withdrawal in each group had to be stated for a point to be awarded.

The funnel plots of the studies were symmetric for HbA1c, fasting glucose and fasting insulin on visual examination. Furthermore, the results of the Egger's test did not support the existence of publication bias for HbA1c (P = 0.46), fasting glucose (P = 0.87), or fasting insulin (P = 0.27).

## Discussion

This meta-analysis of fourteen RCTs with 714 subjects demonstrated that vitamin E supplementation was not associated with the reduction in HbA1C, fasting glucose and fasting insulin. However, significant heterogeneity was detected in all three pooled analyses. Therefore, further subgroup analysis was performed to identify the source of heterogeneity. The subgroup analyses indicated that vitamin E supplementation significantly decreased both HbA1c and fasting insulin in studies with low serum vitamin E concentrations and poorer glycaemic control. Furthermore, larger vitamin E doses and longer study durations also benefited HbA1c and fasting insulin concentrations. Although this meta-analysis failed to identify significant correlations between vitamin E supplements and improvements in glycaemic control, our results suggest that T2DM subjects with low serum vitamin E concentrations or poorer glycaemic control may experience a positive change in glycaemic control after vitamin E supplementation.

Recent mechanistic studies have examined the effects of vitamin E on glycaemic control and provide further evidence for the biological plausibility of these findings. There is now considerable evidence that oxidative stress plays an important role in the glycation of haemoglobin [39] and beta-cell dysfunction [6] in T2DM. Vitamin E, a common antioxidant, suppresses ROS generation in the pancreas and maintains the structural integrity of pancreatic islets in experimental diabetes [8]. Furthermore, there is evidence that vitamins E supplementation inhibits the glycation of haemoglobin, a biomarker for the diagnosis of diabetes in clinical settings, by interrupting glycosylation at an early step in the Maillard reaction [40] or by partially inhibiting the formation of AGEs [11]. Moreover, in addition to its beneficial effects on the preservation of pancreatic beta cell function, vitamin E partially reverses the beta-cell apoptosis caused by oxidative stress [8], [12].

Observational prospective cohorts and case-control studies have been performed to determine the effect of vitamin E supplementation on glycaemic control, although the results are conflicting. Observational prospective cohorts have indicated that low serum vitamin E concentrations are associated with a lower risk of diabetes. A large, 23-year population-based study involving 4304 middle-aged individuals from Finland revealed that, vitamin E intake, including α-tocopherol γ-tocopherol, δ-tocopherol, and β-tocotrienol were significantly associated with a reduced risk of T2DM [9]. In contrast, some RCTs have demonstrated no association between vitamin E supplementation and the risk of T2DM [41]. Case-control trials also demonstrated that vitamin E supplementation exerts beneficial effects on glycaemic control, including serum HbA1c [27], fasting glucose [29] and fasting insulin [30] in T2DM subjects. In contrast, several studies have reported no positive correlations between vitamin E intake and glycaemic control in subjects with T2DM [33].

In this meta-analysis, subgroup analysis was performed on the basis of our predefined variances to identify sources of heterogeneity. It is reasonable to speculate that the treatment effects of vitamin E are influenced by its baseline levels. Subgroup analyses revealed that vitamin E supplementation has a more pronounced effect on HbA1c and fasting insulin in studies with low baseline serum vitamin E concentrations, which is consistent with the findings of an earlier meta-analysis by N. Suksomboon et. al [42]. This increased effect may indicate that extra vitamin E is needed for subjects with vitamin E deficiencies to antagonise oxidative stress, inhibit glycation of haemoglobin and reverse beta cell dysfunction. Our findings also suggest that subjects with poor HbA1c and fasting glucose control would experience the maximum benefit of vitamin E on reducing HbA1c and fasting insulin concentrations, perhaps by controlling excess oxidative stress secondary to hyperglycaemia, inhibiting the glycosylation of haemoglobin, and / or protecting beta cells function in those patients with poor glycaemic control. Subjects with larger doses of vitamin E and longer treatment periods may experience increased benefits of vitamin E supplementation on HbA1c or fasting insulin control. It is possible that shorter T2DM trials may have been insufficient to detect true HbA1c and insulin changes; in addition, smaller doses of vitamin E may not sufficiently elevate vitamin E levels.

The analysis of our secondary outcomes revealed a tendency but no significant improvement in HOMA-IR, postprandial glucose and blood pressure in the vitamin E group compared with controls. In addition, vitamin E supplementation had no significant effect on lipid metabolism, which was consistent with the findings of an earlier meta-analysis by Shekelle PG [43].

The strength of this study was that trials included in this meta-analysis were all RCTs, which allow reliable inferences about causality. In addition, this meta-analysis assessed the effects of vitamin E supplementation on plasma concentrations of fasting glucose and fasting insulin for the first time. Furthermore, subgroup analyses were performed to detect sources of heterogeneity for primary outcomes.

Although we believe that the current meta-analysis provided useful information, some potential limitations should be addressed. First, in this meta-analysis, small trials with limited subjects were collected, which limited the ability to extrapolate the outcomes of this review toward realistic public health recommendations for vitamin E supplementation for glycaemic control in T2DM. Second, there was considerable evidence of trial heterogeneity. Subgroup analyses revealed that vitamin E dosage, trial duration, glycaemic control, ethnic group and serum vitamin E levels may contribute to the variation in trial effects. Therefore, future studies focusing on the effects of different subject characteristics on changes in glycaemic control after vitamin E supplementation are needed to confirm our results. Third, the quality of RCTs included in this meta-analysis was varied. Of the fourteen trials, only one trial was a high-quality study; the other studies were of low quality, which may affect the confidence of this meta-analysis. Publication bias is also a potential limitation. We explored the possibility of publication bias using a funnel plot and Egger's test and found that publication bias was unlikely to have significantly affected our study results. Fourth, our meta-analysis did not recognise a safety margin of vitamin E supplementation because no serious side effects were reported in the included trials. However, concern has been raised as to the safety of high-dose vitamin E supplementation. A meta-analysis by Miller ER demonstrated that vitamin E doses >400 IU per day in patients with chronic diseases may increase all-cause mortality by increasing the risk of prolonged bleeding time and antagonising vitamin K functions in individuals routinely ingesting various drugs [44].

In conclusion, to date, evidence from randomised, controlled trials suggests that vitamin E supplementation is insufficient to decrease serum HbA1c, fasting glucose and fasting insulin concentrations in subjects with T2DM. Subgroup analyses support the hypothesis that vitamin E supplementation significantly reduces HbA1c and fasting insulin concentrations in subjects with low serum vitamin E concentrations and poor glycaemic control. Future large-scale, randomised trials are needed to investigate the effect of vitamin E supplementation on glycaemic control and the potential benefits and toxicities in T2DM subjects.

## Supporting Information

Checklist S1 PRISMA Checklist.
(DOC)

Diagram S1 PRISMA 2009 Flow Diagram.
(DOC)
