# Supplementary material for: Influence of Vitamin E Supplementation on Glycaemic Control: A Meta-Analysis of Randomised Controlled Trials
Source: PLoS One. 2014 Apr 16;9(4):e95008. doi: 10.1371/journal.pone.0095008 (PMC3989270; doi:10.1371/journal.pone.0095008)
Supplement: Diagram S1 — PRISMA 2009 Flow Diagram. (DOC) [file pone.0095008.s002.doc]

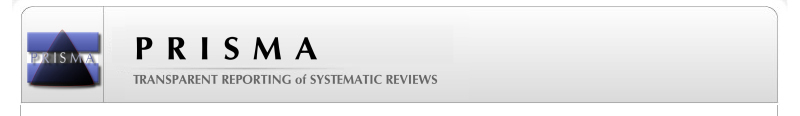
**PRISMA 2009 Flow Diagram**

**Screening**

**Included**

**Eligibility**

**Identification**

Records identified through database searching
(n = 5073 )

Additional records identified through other sources
(n = 0 )

Records after duplicates removed
(n = 4476 )

Records screened
(n =4476 )

Records excluded
(n =4424 )

Full-text articles assessed for eligibility
(n = 52 )

Full-text articles excluded, with reasons
(n = 38 )

Studies included in qualitative synthesis
(n =14 )

Studies included in quantitative synthesis (meta-analysis)
(n =14 )
